# The Mobility Assessment Course for the Diagnosis of Spatial Neglect: Taking a Step Forward?

**DOI:** 10.3389/fneur.2017.00563

**Published:** 2017-10-31

**Authors:** Megan Grech, Tracey Stuart, Lindy Williams, Celia Chen, Tobias Loetscher

**Affiliations:** ^1^School of Psychology, Social Work, and Social Policy, University of South Australia, Adelaide, SA, Australia; ^2^Guide Dogs SA/NT, Adelaide, SA, Australia; ^3^School of Health Sciences, University of South Australia, Adelaide, SA, Australia; ^4^Department of Ophthalmology, Flinders University, Adelaide, SA, Australia

**Keywords:** assessment of neglect, mobility, vision, clinical utility, ecological validity, sensitivity, specificity

## Abstract

Spatial neglect after stroke can be a challenging syndrome to diagnose under standard neuropsychological assessment. There is now sufficient evidence that those affected might demonstrate neglect behavior in everyday settings despite showing no signs of neglect during common neglect tasks. This discrepancy is attributed to the simplified and unrealistic nature of common pen and paper based tasks that do not match the demanding, novel, and complex environment of everyday life. As such, increasing task demands under more ecologically valid scenarios has become an important method of increasing test sensitivity. The main aim of the current study was to evaluate the diagnostic utility of the Mobility Assessment Course (MAC), an ecological task, for the assessment of neglect. If neglect becomes more apparent under more challenging task demands the MAC could prove to be more diagnostically accurate at detecting neglect than conventional methods, particularly as the time from initial brain damage increases. Data collected by Guide Dogs of SA/NT were retrospectively analyzed. The Receiver Operating Characteristic (ROC) curve, a measure of sensitivity and specificity, was used to investigate the diagnostic utility of the MAC and a series of paper and pencil tests in 67 right hemisphere stroke survivors. While the MAC proved to be a more sensitive neglect test (74.2%) when compared to the Star Cancellation (43.3%) and Line Bisection (35.7%) tests, this was at the expense of relatively low specificity. As a result, the ROC curve analysis showed no statistically discernable differences between tasks (*p* > 0.12), or between subacute and chronic groups for individual tasks (*p* > 0.45). It is concluded that, while the MAC is an ecologically valid alternative for assessing neglect, regarding its diagnostic accuracy, there is currently not enough evidence to suggest that it is a big step forward in comparison to the accuracy of conventional tests.

## Introduction

The impact of stroke is devastating. Presently, stroke remains one of the top causes of disability-adjusted life years lost globally (1). A common disability of stroke is spatial neglect, the hallmark symptom being a failure to report or respond to stimuli presented in the contralesional space (2). Importantly, neglect is not attributed to primary sensory or motor defects and is commonly considered to be an attention-related disorder (2). Neglect after stroke affects up to 82% of right hemisphere damaged persons but may also occur after left hemisphere damage (3). The prevalence of neglect is thought to decrease with time, with most recovery happening within the first 12–14 weeks post-stroke before a plateau is reached (4, 5).

The presence of neglect is associated with extended hospital stays (6), and poor functional outcomes and higher requirements for assisted care when likened to stroke victims without the condition (7, 8). While many people recover rapidly, the more persistent symptoms of neglect make it difficult to live independently and safely (9). Neglect increases the person’s risk of accident and injury when crossing roads and having to navigate potentially dangerous objects in the environment (10). Accordingly, the increased need for assistance with daily routines places a significant burden on the families of those affected (11).

To mitigate the harmful consequences of neglect, reliable and accurate symptom detection is key. The most commonly employed clinical tests use paper-and-pencil methods requiring the individual to cancel out static targets (12), to indicate the middle of a line (13), or to copy an object (14). More functional neglect tests assess presence and severity of neglect in daily activities, such as dressing, eating, navigating, and locating familiar items (15–17). However, of the countless neglect tests that are now available, many report low sensitivity (18). Moreover, there is now sufficient evidence that those affected may exhibit neglect behavior despite showing no signs of neglect in common paper-and-pencil tasks (19–32).

An important reason for the relatively low sensitivity of paper-and pencil tasks is their failure to capture everyday demands. Neuropsychological assessments are designed to elicit an optimal performance, whereby distractors, task demands, and task length is controlled and kept to a minimum (33). Such controlled testing situations, however, do not represent everyday life in which patients continuously face novel, dynamic, and complex situations. Indeed, task complexity is a well-established modulator of neglect, and several studies have shown that neglect behavior becomes more apparent with increasing task demands (30, 32, 34).

Failure to detect neglect has important clinical implications. First, undetected neglect may prevent proper access to rehabilitation services. Demonstrating the presence of neglect is a prerequisite of initiating interventions that reduce symptoms and increase independence. Second, a significant number of those affected by neglect may return to their premorbid activities where they put themselves and others at risk in activities of daily living such as driving, road crossing, and the use of dangerous objects/devices (30). Finally, upon assessing the effectiveness of the intervention, there is a risk that the marked improvement in paper-and-pencil task performance might not necessarily translate to everyday life (35). Previous research highlights that a lack of ecologically valid tasks to judge the usefulness of treatments to improve ordinary skills in persons with neglect is a fundamental weakness of *many* clinical neglect trials (35, 36).

In light of the aforementioned issues, a number of alternatives have been proposed. One of these alternatives is the observation of how persons affected by neglect scan the environment while walking a designated course. That is, the individual is asked to walk a standardized Mobility Assessment Course (MAC) and to detect targets located on the corridor walls. The increased task demands and added complexity of walking under multitask conditions have been shown to increase neglect behavior (34). The simplicity of this alternative with high face validity is very appealing as it is easily carried out in a variety of settings, including hospital wards and rehabilitation facilities. Surprisingly, the alternative has not drawn a lot of research interest as of today, nor has there been a thorough investigation of the MACs diagnostic utility as a suitable neglect measure.

The studies that investigated the effectiveness of the MAC as a tool for measuring neglect report encouraging results (37, 38). Both studies demonstrated that neglect participants missed significantly more left-sided targets than controls and that this neglect is also associated with the performance in common neglect tasks. For example, targets missed on the left correlate with performance in the Behavioral Inattention Test (BIT) (37) and the Catherine Bergego Scale (38). The expected correlations provide significant support for the construct validity of the MAC. Moreover, both studies judged the MAC to be an ecologically valid test that is quick to administer and relatively straight forward to implement in clinical settings. Albeit, a complete standardization across different testing times and settings appears less achievable since corridor design and traffic flow are likely to differ in various institutions (38).

The aim of the current study was to expand on the findings of Ten Brink and colleagues by evaluating the diagnostic utility of the MAC paralleled to paper-and-pencil tasks during different stages of stroke recovery (subacute <1 month; chronic > 1 month). The Receiver Operating Characteristic (ROC), was used to evaluate the diagnostic utility of the tasks. Rather than comparing sensitivities of different scores, the ROC-based analysis combines sensitivity and specificity into a single variable, to quantify how accurately a task can discriminate between two states (neglecting and non-neglecting participants). If neglect becomes more apparent under more challenging task demands (31), the MAC could prove to be more efficient at detecting neglect than conventional methods, particularly as the time from initial brain damage increases (28, 29, 39). Moreover, with the MACs improved accuracy, it could offer a more time effective alternative to using large testing batteries since multiple neglect measures will be less necessary.

A secondary aim was to investigate the relationship between basic visual functions and MAC performance. Many stroke survivors report impaired visual abilities (40, 41). While these impairments may be unrelated to neglect, it is conceivable that they could adversely impact the detection of targets in the MAC.

## Materials and Methods

The current study retrospectively analyzed data collected by Guide Dogs SA/NT as a part of the standardized vision assessment of their referred clients. Moreover, 50 healthy participants were prospectively recruited to obtain control data for the MAC. The University of South Australia’s Human Research Ethics Committee approved the study, with all participants signing an agreement to use the results of their vision assessment for the evaluation of Guide Dogs SA/NT assessment procedures.

### Participants

#### Stroke Participants

Clients referred for a vision assessment at Guide Dogs SA/NT between January 1, 2013 and August 31, 2016 were assessed for eligibility. Participants were eligible if they (a) were over the age of 18, (b) were clinically diagnosed with a right-sided cerebrovascular accident (confirmed by medical referral letters), (c) completed a vision assessment on site at Guide Dogs SA/NT by the same expert Orientation and Mobility Instructor (OMI), (d) had satisfactory ability to produce and understand language (assessed by OMI), and (e) were physically able to participate (walking aids and self-propelling wheelchairs permitted).

#### Controls

Fifty healthy age-matched controls came from a convenience sample consisting of volunteer groups associated with Guide Dogs SA/NT.

### Procedure and Tasks

All stroke participants completed a battery of tasks relating to visual function, paper-and-pencil neglect assessments and the MAC.

#### Visual Function Assessment

The visual function assessment comprised visual field testing using the Neuro Vision Technology (NVT) System and confrontation testing. The NVT system consists of the NVT scanning device (a light bar consisting of two rows of 10 colored lights, displayed on a horizontal panel placed at eye level approximately 30 cm from the client, and extending approximately 80 cm either side of central fixation) and computer software utilizing a standardized presentation of lights to determine the presence and degree of field loss (37). A secondary confrontation visual field test (Donders’ test) was used to provide additional information regarding the presence of gross field loss. Visual reading acuity was measured using Dr. Alan Johnston’s logMAR chart and low contrast reading chart. For overall contrast sensitivity, the Mars Letter Contrast Sensitivity Test (log contrast sensitivity) was employed.

#### Paper-and-Pencil Neglect Assessments

Two paper-and-pencil tasks were previously administered to the participants during their vision assessment. These included the Star Cancellation and the Line Bisection, both of which are subtests of the BIT (42). All tasks are on an A4 piece of paper, centered to the participant. Participants were instructed to avoid leaning to one side and to avoid adjusting the position of the paper during the assessment. However, there was no restriction of head and eye movements. Time pressure was, also, not imposed.

The Star Cancellation (43) consists of 56 small stars (the targets), 52 larger stars, 12 letters, and 10 short words in pseudo random order. Firstly, the administrator demonstrates by crossing out two of the small stars located in the middle of the page. The participant is then instructed to locate all remaining small stars (27 on the left and 27 on the right). The maximum score is 54. Previous research suggests neglect is evident in scores ranging from 44 to 51 (44–46). In the current study, we compared both cutoffs for star score (51 and 44) by computing the sensitivities and specificities for each cutoff with reference to the other neglect tasks. To assess the presence of a lateralizing deficit the star ratio, dividing the number of stars canceled in the left column by the number of stars canceled in the right, was computed. Values close to 0.50 indicate a symmetrical performance. Scores that range between 0 and 0.46 indicated neglect (44, 47).

In the Line Bisection, the participant is presented with three horizontal 8 inches (204 mm) lines offset in a staircase fashion (42). The extent of each line is pointed out to the participant who is then instructed to estimate the middle of each line. Deviations from the midpoint drawn by the participant to the actual midpoint for each line are measured. Scores ranging between 0 and 3 for each line were derived using the BIT template. A smaller score indicates that a mark is placed further from the midline. The maximum score is 9. Scores less than 6.5 indicate the probability of neglect (42).

#### Mobility Assessment Course

**Figure 1:**
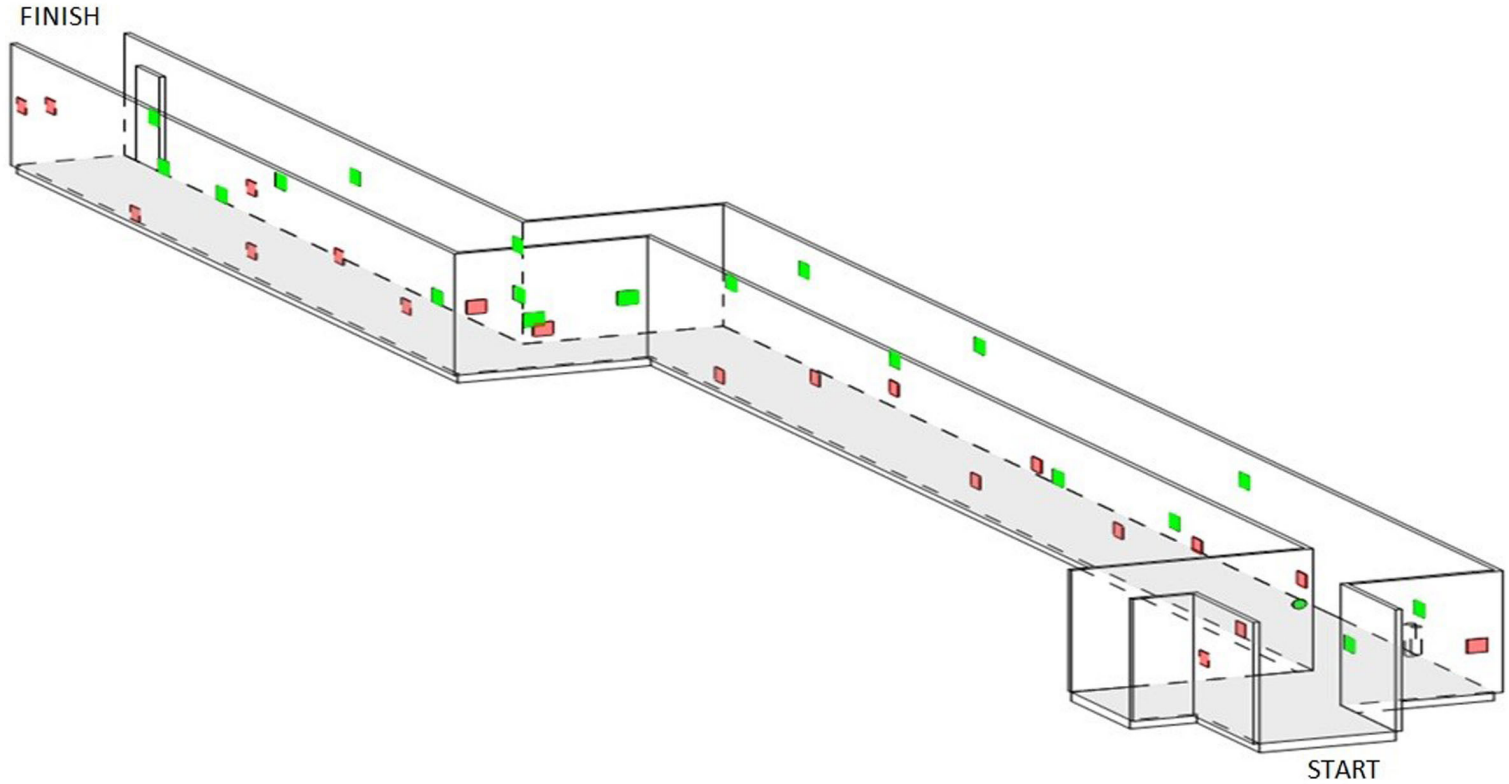
A schematic 3D model of the Mobility Assessment Course situated at Guide Dogs of SA/NT. Red targets are corresponding to the left wall. Green targets are corresponding to the right wall.

The MAC, located at Guide Dogs SA/NT and based on Verlander, Hayes (37), is a standardized route spanning 43 m in length (Figure 1). There is one sharp turn in the middle of the course indicated by an arrow at the wall. Pedestrian traffic is hardly present. However, the corridor is readily accessible to all Guide Dogs SA/NT staff. Along the corridor, 40 targets (20 on each side) are located on the walls at varying heights, ranging in size (10–20 cm), shape (squares, rectangles, circles, and stars), and color (yellow, blue, orange, pink, silver, and gold). Consistent scanning was required as targets were occasionally obstructed from view (Inside windowsills and behind fire hydrants) until the participant reached the target.

An example of the targets is shown to each participant at the beginning of the course. The instruction was to move through the course at a leisurely pace and to find all targets, so as to represent a dual-task (walking and visual search). The OMI followed, so as not to hinder the participant’s mobility, while the participant pointed to each target as it became visible. The following outcomes were recorded: the total percentage of targets found and the asymmetry score, indicated by the difference between the number of targets found on the left and the right sides. A lower total score indicates a poorer performance. A higher asymmetry score, indicating more targets located on the right than left sides, reflects a poorer performance.

### Statistical Analyses

Participants were grouped based on a 2-h vision assessment run by an experienced OMI. There is a lack of a reliable gold standard for the diagnosis of neglect (48), thus, as in Rengachary et al. (39), the clinical diagnosis of left neglect was the criterion standard used in defining the presence of neglect. A single OMI with 15 years’ experience in vision and neglect assessments made the diagnosis. Participants who were deemed to have neglected based on this evaluation formed the neglect group. Participants without a neglect diagnosis were referred to the non-neglect group. The groups were then further subdivided into subacute (<1 month) and chronic (>1 month) stages of stroke recovery.

The diagnostic utility of each task was evaluated using the Receiver Operating Characteristic (ROC) curve (49, 50). Often, specificity is neglected when assessing the diagnostic accuracy of neglect assessments. The ROC analysis combines both sensitivity and specificity to quantify how accurately a test can separate the tested groups into neglecters and non-neglecters. The sensitivity of a clinical test refers to the ability of the test to identify the participants with neglect correctly. A test with 100% sensitivity/100% specificity, suggests that the test identified all participants with neglect (sensitivity) and without neglect (specificity) correctly (51).

The ROC-based analysis (52) trade-offs between sensitivity (true positives) and specificity (false positives). In the ROC curve, the true positive rate is plotted against the false negative rate for different cutoff points. Each point on the ROC curve represents a single cut-off for the plotted pair (sensitivity, 1-specificity) corresponding to a chosen threshold. The area under the ROC curve (AUC) represents a measure of the test’s discriminatory power. The higher the score, the greater discriminatory ability of the test [i.e., the true positive rate is high and the false positive (1-Specificity) rate is low]. The AUC can range from 0.0 to 1.0. Interpretation of AUC values are such that a value of 1.0 suggests perfect discriminatory abilities, that is all participants with neglect, and without neglect, are classified accordingly, 0.9–0.99 has outstanding discriminatory abilities, 0.8–0.89 has excellent discriminatory abilities, 0.7–0.80 has acceptable discriminatory abilities, 0.51–0.69 has poor discriminatory abilities, and a value of 0.5 suggests that the test is “no better than chance” at discriminating neglect participants from non-neglect participants (53).

To compare the diagnostic accuracy of the AUC at different levels, we used the methods of DeLong et al. (54) and Hanley and McNeil (51). For independent sample analyses, such as the comparisons between subacute and chronic groups, any significant differences were evaluated using the methods of Hanley and McNeil (51). For differences in same sample comparisons between tasks, DeLong et al.’s (54) methodology was employed. For all multiple comparisons, the chosen adjusted alpha level was 0.01. Statistical analyses were performed using MedCalc software for Windows, version 9.3.2.0 (MedCalc Software bvba, Mariakerke, Belgium) and IBM SPSS Statistics for Windows, version 20.0 (IBM Corp., Armonk, New York, NY, USA).

The same threshold for neglect as in Azouvi et al. (21) was applied for the MAC, such that any participant scoring poorer than the fifth percentile of the control group was considered to be affected by neglect.

We investigated the relationships between performance at the MAC, paper-and-pencil tasks, and the visual function measures with a series of Spearman correlations. The statistical significance was set at 0.05. An *r* of 0.1 was considered a small, 0.3 a medium, and 0.5 a large correlation (55).

## Results

Overall, this study included 67 stroke survivors and 50 healthy control participants (Table 1). There were no significant differences between the right hemisphere damage and control groups in relation to age, *t* = 0.782, *p* = 0.436. The 50 healthy controls completed the course providing participant cutoffs for indicating the presence of neglect. Control participants found on average 84.70% (SD 7.31) of the targets located on the MAC with an asymmetry score of −2.15 targets (SD 4.68). Applying a fifth percentile cutoff criterion for neglect (21) the threshold for neglect was an asymmetry score of >+6.13% and a total target score of <71.30%.

**Table 1:** Group demographics divided by subacute and chronic stages of recovery for neglecters and non-neglecting right hemisphere damage participants.

	Subacute (<1 month)		Chronic (>1 month)		
	Neglecters, *N* = 11	Non-neglecters, *N* = 17	Neglecters, *N* = 20	Non-neglecters, *N* = 19	Controls (*n* = 50)
Gender (Male/Female)	8/3	11/6	15/5	13/6	16/34
Age in years, M (SD)	73.82 (7.20)	64.88 (14.54)	67.90 (12.29)	58.37 (18.33)	63.40 (11.95)
Days since stroke, M (SD)	20.91 (8.23)	21.53 (6.92)	178.10 (301.12)	399.89 (1140.25)	–
Visual field defect (yes/no)	10/1	11/6	16/4	14/5	–
Mobility					
Independent no aids Independent (walking cane) Wheelchair	8 3 0	17 0 0	18 0 2	18 1 0	50

As shown in Table 2, the MAC identified more neglect cases than any other paper-and-pencil task. The total number of targets found was a better predictor of neglect than asymmetry scores. The total and asymmetry scores on the MAC exposed a considerable amount of false positives compared to both paper-and-pencil tasks, misclassifying more individuals as affected by neglect when the clinical diagnosis suggested otherwise. Importantly, while the paper-and-pencil tasks showed remarkable specificity values, the low sensitivity meant a high number of positive neglect cases were overlooked.

**Table 2:** Number of true/false negative/positive calculations for the Star Cancellation using the star ratio and two laterality indexes (<44, <51), the Line Bisection—deviation score, and the MAC total score and asymmetry cutoffs derived from the control data.

	*N*	Cutoff point	Mean (SD)	% beyond cutoff point	True positives (sensitivity)	False positives	True negatives (specificity)	False negatives
**Star cancellation**								
Total	66	<51	50.95 (5.84)	22.4	13 (43.3%)	2 (5.6%)	34 (94.4%)	**17 (56.7%)**
		<44	50.95 (5.84)	10.4	6 (20%)	1 (2.8%)	35 (97.2%)	24 (80%)
Star ratio	66	<0.46	0.48 (0.06)	12.1	8 (26.7%)	0 (0%)	36 (100%)	21 (73.3%)
Line Bisection, deviation score	59	<6.5	7.95 (2.15)	14.9	10 (35.7%)	0 (0%)	31 (100%)	18 (64.3%)
**MAC**								
Total	67	<71.3%	73.95 (15.00)	**50.7**	**23 (74.2%)**	11(30.6%)	25 (69.4%)	8 (25.8%)
Asymmetry (R minus L total)	67	>+6.13%	2.40 (10.71)	31.3	12 (38.7%)	9 (25%)	27 (75%)	19 (61.3%)

*The number of participants completing each task differs as the availability of test data varied*.

**Figure 2:**
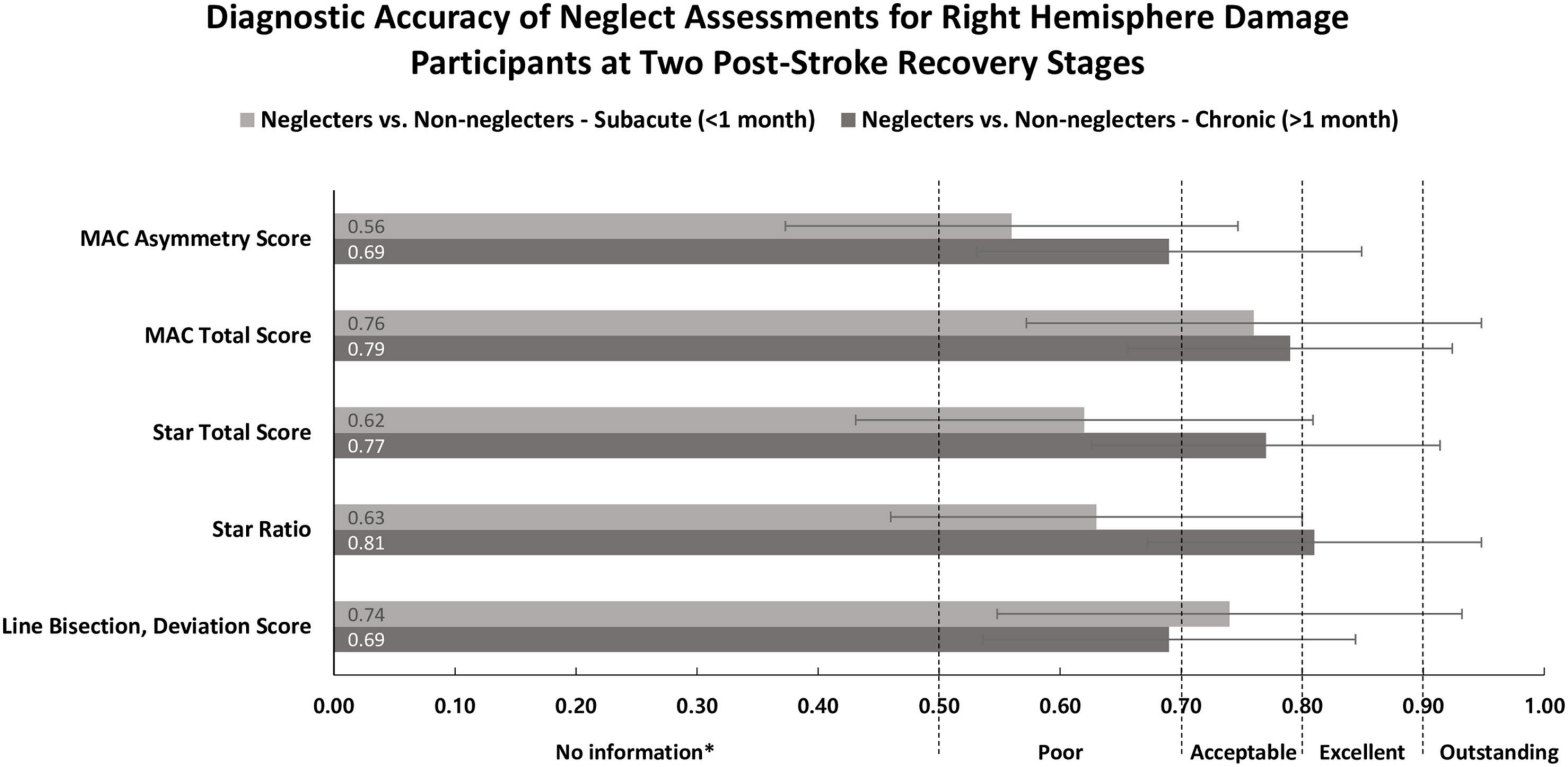
Area under the ROC curve (*x*-axis) and neglect measures (*y*-axis) for participants in subacute (<1 month) and chronic (>1 month) recovery stages. Classification terms (poor, acceptable, excellent, and outstanding) refer to the discriminative abilities of each outcome. Error bars represent 95% confidence intervals. Abbreviations: R, right; L, left; MAC, Mobility Assessment Course. *Test provides no useful information.

Figure 2 displays the AUC as a measure of the diagnostic utility of each task. To investigate the performance as a function of time post stroke, the AUC was computed at two stages of stroke recovery, subacute (<1 month) and chronic (>1 month). The AUC values indicated that no single test was considered to have outstanding accuracy in distinguishing a neglecting participant from a non-neglecting participant. When considering the tests discriminative abilities, the majority of the AUC values fell in the “poor” to “acceptable” range. As per *post hoc* tests, no significant differences existed between tests at subacute or chronic stages of recovery (*p* > 0.12), or between subacute and chronic groups for individual tasks (*p* > 0.45).

All MAC measures were significantly related to the Star Cancellation ratio (Table 3). Similarly, all measures on the MAC, except for asymmetry, showed moderate to large correlations with the Line Bisection deviation score.

**Table 3:** Spearman correlations between MAC, Star Cancellation, Line Bisection, contrast sensitivity, and visual acuity.

		MAC targets found		

Outcome	*N*	Total	Contralesional	Asymmetry
Star Cancellation, star ratio	66	0.40**	−0.42**	−0.27*
Line Bisection, deviation	60	0.58**	0.48**	−0.07
Visual acuity	66	−0.11	−0.14	0.08
Contrast sensitivity	66	0.13	0.05	−0.07

*The number of participants completing each task differs as the availability of test data varied*.

*Correlation is significant at the 0.05 level.

**Correlation is significant at the 0.001 level (two tailed).

*MAC, Mobility Assessment Course*.

## Discussion

The main aim of the current study was to evaluate the diagnostic utility of the MAC compared to a series of paper-and-pencil neglect tests during different stages of stroke recovery. The hypotheses that the MAC measure with greater task demands, relevancy, and transparency could improve detection of neglect, compared to the conventional tests, particularly at more chronic stages of recovery, were not supported. The 95% confidence intervals of the AUC values overlapped across the different test scores and recovery stages. There were no statistically discernable differences between tasks at subacute or chronic stages or, between subacute and chronic groups for individual tasks. These results suggest, irrespective of time since brain damage, the MAC is just as accurate at detecting neglect as the tasks under question here.

Our results are in contrast to the abundance of literature suggesting a superiority of functional and more demanding tasks over conventional paper-and-pencil neglect tasks (21, 26, 27, 31, 56, 57). An explanation for the discrepancy is that the current study investigated test accuracy by combining the task’s sensitivity and specificity into one measure. Indeed, if we only assess the sensitivity of the test, i.e., their ability to detect neglect (true positives), the MAC showed the highest sensitivity values (74.2%). The symptom detection skills of the Star Cancellation (43.3%) and Line Bisection tests (35.7%) are less comparable. However, the MAC’s high sensitivity is at the expense of a low specificity, as such, the MAC (based on total targets located) falsely implicated 30.6% of our participants without neglect as affected by neglect. In comparison, the Star Cancellation falsely diagnosed only 2.8% of the sample as affected by neglect.

The different sensitivity and specificity values of the MAC and the conventional tests are, therefore, a likely reason that the combined inspection of these two values in a ROC curve does not reveal statistically significant differences between the tests. Thus, at the very least our results highlight the importance of taking into account sensitivity and specificity when evaluating the diagnostic utility of a test. It is noteworthy that the large body of literature on neglect assessments focuses on sensitivity testing, and thereby neglects specificity (58–61), as such, crucial information is missing that adequately judges the true diagnostic accuracy of these tests.

From a clinician’s point of view, it might be more acceptable for an assessment tool to be less specific, than less sensitive, since the main purpose of the clinical assessment is to uncover symptoms. The high sensitivity of the MAC has an apparent advantage in circumstances in which significant remaining impairments are monitored safely. For example, the discovery of a contralesional deficit in attention which would affect driving ability and subsequently avoid crashes. What is significantly riskier is a missed diagnosis that denies the person access to vital assisted care or rehabilitation. As a result, the risk of accidents and injuries increases, particularly if a suspended license is reinstated or the individual returns to work.

The AUC values indicate that no single test score has outstanding accuracy in distinguishing a non-neglecting participant from a neglecting participant. The majority of the measures fell into the “poor” to “acceptable” range. Just like the standard practice in current neglect assessments, the implications of the tests suboptimal diagnostic accuracies are clear cut; to guarantee the correct diagnosis of all persons affected by neglect, the administration of a battery of neglect tests is necessary. In other words, the idea that the MAC could potentially justify the reduction of tests required for a neglect diagnosis was not supported. It is important to keep in mind that neglect is a heterogeneous syndrome manifesting in many different ways (62). It is, therefore, unlikely that we will capture neglect in its many forms with just one task. Nevertheless, a quick assessment of neglect (i.e., fewer tasks) would be advantageous since it makes room for further neurological testing while reducing the risk of exhaustion in the participant.

The MAC measures (total and contralesional targets found) were significantly related to performances in the conventional neglect tests. The observed medium to large effect sizes is similar in size to previously reported relationships between the performances in the MAC and standard neglect tests (37, 38). The finding of these relationships across three different studies each with different MAC settings and designs points to the MACs high construct validity and may help to further establish the MAC as an ecologically valid alternative for assessing neglect.

An important novel aspect of this study was the assessment of the effects of basic visual functions on the performance in the MAC. We found no relationship between the patients (corrected) visual acuity, contrast sensitivity, and their ability to spot targets during the MAC. These results need further confirmation with larger and more diverse samples, but low vision impairments commonly found in stroke survivors may not significantly affect the diagnostic properties of the MAC. If this holds true, then this further adds to the validity of the MAC.

An interesting, yet still unanswered question, is whether test accuracy is modulated by the presence or absence of visual field defects. Only a small proportion of participants (15%) were without a visual field defect in this study; we, therefore, were unable to assess its effects on the MAC reliably. Buxbaum et al. (59) found that a virtual reality program (VRLAT) was equally as likely to categorize those with and without visual field defects as being affected by neglect. The VRLAT has many similarities with the MAC. Therefore, the MACs abilities to differentiate between those with and without field defects are promising. Future research is essential to evaluate this likelihood further.

In sum, validating previous studies (37, 38), we conclude that the MAC is an ecologically valid alternative for assessing neglect. Regarding its diagnostic accuracy, there is currently not enough evidence to suggest that it is a big step forward in contrast to the accuracy of more commonly used tasks. However, the high sensitivity of the MAC has an apparent advantage when screening neglect and positive results can be further investigated due to the tests relatively low specificity. Moreover, the MAC is likely to be an insightful exercise for people affected by neglect. The participant can repeat the course allowing the clinician to make visible neglected targets and to provide feedback on performance characteristics, such as walking speed or visual scanning behavior. The MAC does highlight self-awareness in contralesional deficits in a more practical sense and, therefore, is considered here to be a step in the right direction.

## Ethics Statement

The University of South Australia’s Human Research Ethics Committee granted approval for the study, with all participants signing an agreement to use the results of their vision assessment for the evaluation of Guide Dogs SA/NT assessment procedures.

## Author Contributions

TL, MG, LW, and TS conceived and designed the study. MG and TS collected data. TL and MG analyzed the data. All authors were involved in the interpretation of the data, writing and drafting of the manuscript, and overlooked the final manuscript before submission.

## Conflict of Interest Statement

The authors declare that the research was conducted in the absence of any commercial or financial relationships that could be construed as a potential conflict of interest.
